# Reviewed article: Lopes MS, Sathler IZ, Soares CSJ, Ferreira NL, Lopes ACS. Distance professional qualification to promote adequate and healthy eating: factors associated with dropout. Epidemiol Serv Saude. 2025:34;e20240338.

**DOI:** 10.1590/S2237-96222025v34e20240338.a

**Published:** 2025-08-08

**Authors:** Isabella da Motta Passos

**Affiliations:** Universidade do Estado do Amazonas

**Table:** 

Rating	Excellent	Good	Average	Below average	Poor
Interest			✓		
Quality		✓			
Originality			✓		
Overall		✓			

**Table:** 

Questionnaire	Yes	No	Not applicable
Does the manuscript contain new and significant information to justify publication?	✓		
Does the Abstract (Summary) clearly and accurately describe the content of the article?	✓		
Is the problem significant and concisely stated?	✓		
Are the methods described comprehensively?	✓		
Are the interpretations and conclusions justified by the results?	✓		
Is adequate reference made to other work in the field?	✓		

## Manuscript Structure

Length of article is: Adequate

Number of tables is: Adequate

Number of figures is: Adequate

Please state any conflict(s) of interest that you have in relation to the review of this paper (state “none” if this is not applicable): None

## Comments

O manuscrito intitulado “Qualificação profissional à distância para promoção da alimentação adequada e saudável: fatores associados à evasão” possui conteúdo dentro do escopo da RESS.

O manuscrito está bem estruturado. Foi descrito de forma mais suscinta a idealização da problemática na introdução e a metodologia foi colocada de forma satisfatória. O manuscrito possui bastante dados, que também foram descritos de forma direta e suscinta, faltando descrever melhor as características entre dos grupos das figuras 2.2 e 2.3 (COVID-19 interferiu e não interferiu na evasão do curso). A discussão foi detalhada, procurando justificar cada tópico estatisticamente significativo dos resultados apresentados. Minha única sugestão é procurar saber se em outros artigos citados (7,12,13), os motivos da evasão foram semelhantes ao deste manuscrito. E no final, a conclusão do estudo é pertinente ao sugerir que medidas de apoio institucional são permitidas para superar as barreiras identificadas.

Apesar do manuscrito não trazer dados inédito ou inovadores, possui o conteúdo de relativa significância, com material bem estruturado e descrito. Portanto, a minha recomendação é aceitar o manuscrito, sendo as poucas observações feitas para enriquecer o material é de caráter facultativo.

